# The Increased Sex Differences in Susceptibility to Emotional Stimuli during Adolescence: An Event-Related Potential Study

**DOI:** 10.3389/fnhum.2017.00660

**Published:** 2018-01-12

**Authors:** Jiemin Yang, Shu Zhang, Yixue Lou, Quanshan Long, Yu Liang, Shixue Xie, Jiajin Yuan

**Affiliations:** The Laboratory for Affect Cognition and Regulation (ACRLAB), Key Laboratory of Cognition and Personality of Ministry of Education (SWU), Faculty of Psychology, Southwest University, Chongqing, China

**Keywords:** adolescence, pubertal development, emotional sensitivity, event-related potentials, sex difference

## Abstract

The present study investigated how pubertal development and sex interact to influence humans’ emotion susceptibility during adolescence. Event-related potentials were recorded for highly emotional, mildly emotional and neutral stimuli in positive and negative blocks, when 73 adolescents (36 pre-/early pubertal students, 19 boys, 10–12 years old; 37 mid-/late pubertal students, 18 boys, 11–13 years old) performed an implicit emotion task. Behavioral analysis showed higher positive mood ratings for pre-/early compared to mid-/late pubertal subjects, irrespective of sex and block. ERP analysis demonstrated increasing Late Positive Potential (LPP) amplitudes from neutral, Mildly Positive (MP) to Highly Positive (HP) stimuli in pre-/early pubertal, but not in mid-/late pubertal adolescents. However, girls exhibited higher P3a amplitudes during mid-/late relative to pre-/early puberty for negative stimuli irrespective of intensity; while this puberty effect was absent in boys. In addition, girls compared to boys exhibited a more pronounced LPP enhancement effect for Highly Negative (HN) stimuli and a lower threshold of responding to negative stimuli in P3b amplitudes, regardless of puberty. These results suggest that, though there is a puberty-independent sensitivity to negative stimuli in girls relative to boys, puberty selectively intensifies girls’ attention bias for negative stimuli and reduces experiential sensitivity to positive stimuli in both sexes. The implication of these results for the sex-related psychopathology during adolescence were discussed.

## Introduction

Puberty, as a turning point of adolescent period, is accompanied by prominent physiological (e.g., sex hormone changes and menstruation), physical (e.g., secondary sex characters and height) and psychosocial (e.g., gender role stereotyping) changes (Patton and Viner, 2007; Blakemore et al., 2010; Marceau et al., 2011; Guyer et al., 2014). Each of them has been considered a potential risk factor for adaptation stress and affective disorder during adolescence (Hankin and Abramson, 2001; Ge et al., 2003; Spear, 2009; Duke et al., 2014). In fact, epidemiological studies have consistently shown a higher prevalence of emotional disorders in adolescents relative to children, such as depression, panic disorder, social anxiety, obssessive-compulsive disorder and so on (Hankin, 2006; Patton and Viner, 2007), and this prevalence is higher in girls than in boys (Kessler et al., 1993; Nolen-Hoeksema and Girgus, 1994; Hankin and Abramson, 2001).

However, the mechanisms underlying this phenomenon have not been sufficiently studied. A number of observational studies have suggested a couple of psychosocial factors that contributed to increasing incidence of emotional disorders in adolescence, such as gender role intensification (Aubé et al., 2000), body image concern (Hankin and Abramson, 2001), parent-offspring conflicts (Laursen et al., 1998), interpersonal stress (O’Shea et al., 2014) and academic stress (Quach et al., 2015). While these studies have found that these factors are associated with affective disturbances, and these conclusions were only drawn from the observation designs and psychometric data, such as ICD-10, SCL-90, etc. (Bülow et al., 2002; Vicente et al., 2012; Wesselhoeft et al., 2015). Though observational design is important in showing links between potential contributors and mental health, the lack of experiment manipulation made it hard to answer by causation how puberty and sex interact to influence adolescents’ susceptibility to affective disturbances. Previous studies suggest that the emotional susceptibility was closely related with affective disturbances (Hofer et al., 2006; Yuan et al., 2009), and this susceptibility is embodied by one’s brain sensitivity to emotional stimuli (Carretié et al., 2004; Hofer et al., 2006; Williams and Gordon, 2007; Yuan et al., 2009). In this regard, it is necessary to design an experiment exploring the interactive effects of sex and puberty on brains’ susceptibility to emotional stimuli. However, currently few studies have directly investigated this issue, despite existence of several relevant studies.

For instance, using startling EMG activity for eyeblink as an index of defensive motivation, Quevedo et al. (2009) showed that the mid-/late pubertal adolescents showed enhanced startle reflex amplitudes compared to pre-/early pubertal adolescents, irrespective of picture valence. This suggests that pubertal development is linked with enhanced defensive motivation, which predicts greater levels of fear and anxiety (Pine et al., 1998). However, the lack of behavioral and brain activity measures made it unable to depict a comprehensive profile of how emotional sensitivity varies across puberty and sex. In addition, Fujisawa and Shinohara (2011) investigated sex differences in the recognition of emotional prosody in late childhood and adolescence, and the authors observed that girls were more sensitive to sad and happy prosodies than boys in adolescence but not in childhood. However, this study used an affect recognition task that measured one’s recognition of facial expressions, leaving one’s natural emotional reaction to evocative stimuli undetermined. Moreover, using functional magnetic resonance imaging, Hardee et al. (2017) observed reduced amygdala and precentral gyrus activation for negative vs. neutral words with age increase (ranging from 8.5 to 17.6 years) in males but not in females, which was coupled with age-related increase in internalizing symptomatology experiences for females but not for males. More relevant to the current study, using a cross-sectional design and the time-frequency analysis of EEGs induced by negative pictures, a prior study in our lab has showed that pubertal transition was associated with enhanced gamma oscillations for negative pictures in girls but not in boys, in line with the epidemiological reports of increased prevalence of affective disorders in girls than in boys after entry into puberty (Yuan et al., 2014). Since gamma oscillatory activities were proved as an index of the emotional arousal effect (Bastiaansen and Hagoort, 2006; Balconi and Lucchiari, 2008; Balconi et al., 2009), this result suggests that pubertal development has enhanced girls’ emotional arousal level for negative pictures compared with boys. However, this work did not include positive stimulation, leaving it unknown how positive emotional sensitivity varies across puberty and sex.

Thus, the present study explores how puberty and sex interact to influence the humans’ susceptibility to emotionally negative and positive stimuli, using both behavioral (e.g., experiment-induced mood) and event-related potentials measures. To better assess emotional susceptibility in the behavioral level, we used a block-wise design wherein only emotional stimuli of a specific valence (positive or negative) were presented in a given block. Then, self-ratings of mood were collected before and after either experimental block, to examine how either experimental procedure alters one’s mood and how this modulation varies with puberty and sex. On the other hand, in neurophysiological levels, we varied the valence strength of emotional stimuli in either category since emotion-related individual differences are manifested by both response magnitude differences and different threshold of emotion induction (Yuan et al., 2009, 2012; Lou et al., 2016; Lu et al., 2016). In order to increase ecological validity of emotional induction, we used an implicit task wherein emotional stimulus was presented infrequently and unpredictably, and emotional assessment of the stimulus was not overtly requested (Yuan et al., 2009; Lou et al., 2016).

On the other hand, emotion-related gender differences were mainly manifested by three aspects in prior studies, from attention allocation (Campanella et al., 2004; Yuan et al., 2009); cognitive evaluation (Rhudy and Williams, 2005; Maffei et al., 2015) to late emotional arousal/experiences (Maffei et al., 2015). For example, females, instead of males, probably allocated more attentional resources to the Mildly Negative (MN) stimuli, a processing step that has been shown to be represented by centrally-peaking N2 or P3a in brain potentials during an oddball task (Delplanque et al., 2005; Yuan et al., 2009). In particular, P3a activity was proved as involuntary attention orientating to biologically important, salient stimuli (Friedman et al., 2001; Delplanque et al., 2005; Yuan et al., 2008). In addition, it has been shown that women tend to give a more negative evaluation to ambiguous emotional pictures compared with men (Krohne and Hock, 2008), and this evaluative processing is often illustrated by P3b activity when ERP measures are used (Ito et al., 1998; Delplanque et al., 2005). Also, it has been shown that the same threat cues, like negative film clips, elicited enhanced emotional arousal in women than in men (Rhudy and Williams, 2005; Maffei et al., 2015), and the emotional arousal has been established to covary with the amplitudes of Late Positive Potentials (LPPs) in ERP (Moser et al., 2006; Foti and Hajcak, 2008; Krompinger et al., 2008). Based on these considerations, the current study hypothesized that sex and puberty differences in susceptibility to emotional stimuli would be reflected by attention allocation (N2/P3a), evaluative processing (P3b) and later emotional arousal/experience (LPP) components.

As prior studies have shown that more advanced pubertal status is associated with increased incidence of various affective disorders and this modulation is more pronounced in girls (Hankin and Abramson, 2001; Hankin, 2006), we predict that mid-/late relative to pre/early pubertal subjects would show more pronounced emotion effects for negative and less pronounced emotion effects for positive stimuli in self-rated mood and in attentive (P3a), evaluative (P3b) or experiential (LPP) processing stages, and these pubertal effects would be more robust in girls than in boys. Additionally, accumulating evidences have shown that positive and negative affects are functionally independent in assessing one’s susceptibility to affective disturbances (Larsen and Ketelaar, 1991; Crawford and Henry, 2004; Ding et al., 2015). For instance, the reduced positive affect is a more sensitive predictor for depression compared to the increased negative affect (Dyck et al., 1994; Jolly et al., 1994; Crawford and Henry, 2004), while anxiety is linked with the increase of negative affect and seldom changes of positive affects (Dyck et al., 1994; Jolly et al., 1994). Hence, the current study examined the sex and pubertal effects in sensitivity to positive and negative stimuli, separately, to depict a unique profile for positive and negative emotion varying as a function of sex and pubertal development.

## Materials and Methods

### Subjects

As paid volunteers, 36 pre-/early pubertal students aged in 10–12 years (19 boys, *M* = 11.03, SE = 0.09) and 37 mid-/late pubertal students aged in 11–13 years (18 boys, *M* = 11.73, SE = 0.11) from local primary/middle schools participated in the experiment. All the subjects were sampled randomly and were screened by measuring the Pubertal Development Scale (PDS; Petersen et al., 1988). The PDS is a 4-point and 5-item self-report questionnaire that is widely used for pubertal status measurement (Petersen et al., 1988; Earls et al., 2000). To be specific, the items of growth spurt, body hair development and skin changes are assessed in both genders. Besides, boys were asked to report another two items about facial hair growth and voice change, while the girls’ additional items are breast development and menarche.

Using the grouping standard recommended by previous studies (Quevedo et al., 2009; Forbes et al., 2010, 2011), we classified these subjects into the pre-/early pubertal group (*M*_PDS_ = 3.83, SE = 0.11) and the mid-/late pubertal group (*M*_PDS_ = 7.05, SE = 0.22). In detail, girls scoring 3 or 4 were sorted into the pre-/early group while the others were sorted into the mid-/late group. Boys scoring 3, 4 or 5 were sorted into pre-/early group and the others into the mid-/late group. Nobody scored on 12 (pubertal development completed) in both genders. Boys and girls in either group were matched in age and pubertal scores except for the age of the mid/late sample (Table 1). The analyses of variance (ANOVA) of age with sex and puberty as predictors showed a significant interaction effect (*F*_(1,69)_ = 8.72, *p* < 0.01), with similar ages recorded for boys and girls in pre/early sample (*p* = 0.76) but older age for boys vs. girls in the mid/late sample (*p* < 0.001). As boys usually start pubertal development later than girls (Attallah, 1994; Papadimitriou, 2001; Papadimitriou and Chrousos, 2005; Ferrández et al., 2009), the result of sex difference in the age of mid-/late group showed in Table 1 is understandable.

**Table 1:** The means and standard deviations of ages and the pubertal development scale (PDS) scores for each group.

	Pre-/early puberty			Mid-/late puberty		
	Boys (*N* = 19)	Girls (*N* = 17)	Diff.	Boys (*N* = 18)	Girls (*N* = 19)	Diff.
Age	11.00 ± 0.67	11.06 ± 0.43	*t*_(34)_ = −0.31, ns	12.11 ± 0.58	11.37 ± 0.60	*t*_(35)_ = 3.83, *P* < 0.01
PDS	3.84 ± 0.83	3.82 ± 0.39	*t*_(34)_ = 0.08, ns	6.72 ± 0.90	7.37 ± 1.64	*t*_(35)_ = −1.48, ns

**Figure 1:**
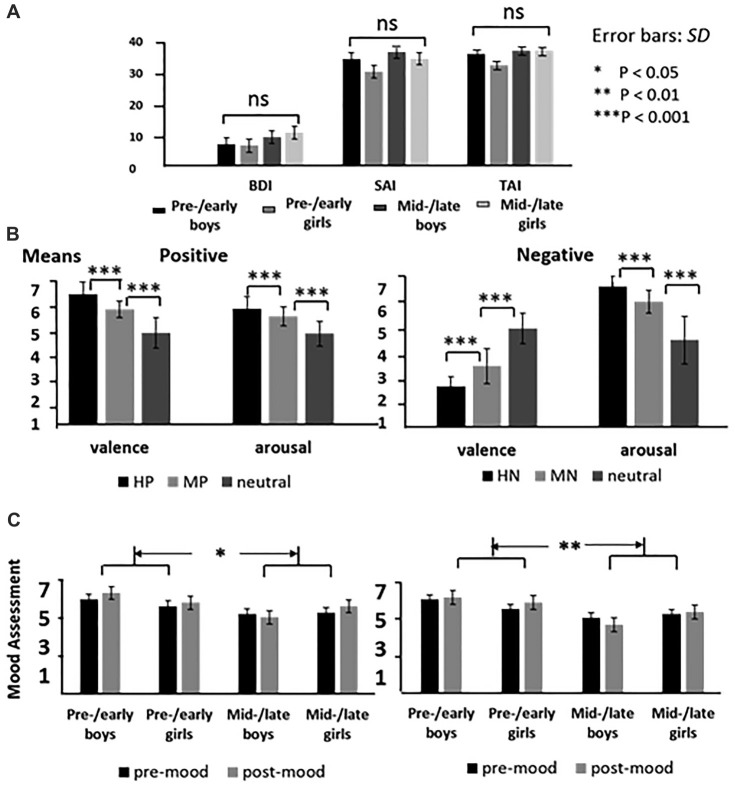
The means of emotional inventory scores for each group** (A)**; the means of valence and arousal for highly emotional, mildly emotional and neutral picture sets in positive and negative blocks **(B)**, and the means of the mood assessment for each group before and after the experiments **(C)**.

All these subjects were asked to finish the Beck Depression Inventory (BDI; Beck, 1978), and the State-Trait Anxiety Inventory (STAI; Spielberger et al., 1983). The ANOVA of these inventory scores with sex and puberty as two predictors showed no significant main or interaction effects (all *p* > 0.30), suggesting that the four samples are similar in the pre-experiment emotional traits (Figure 1A). All the subjects were right-handed, with normal/corrected-to-normal vision and no history of major psychiatric or neurological disorders. The subjects and their guardian have both signed an informed consent form before the experiment. The study was approved by the local Review Board for Human Participant Research, and the experimental procedures were in accordance with the ethical principles of the 1964 Declaration of Helsinki (World Medical Organization, 1996).

### Stimuli

The present study adopted a modified oddball paradigm which included two blocks (positive and negative) of 400 trials each. Each block was divided into four sessions of 100 trials, each of which consisted of 55 standard stimuli and three conditions of 15 deviant stimuli. All the deviant pictures were selected from the Chinese Affective Picture System (CAPS), and the frequent standard stimulus is a picture of a cup. The deviant stimuli were grouped as Highly Positive (HP), Mildly Positive (MP) or neutral in positive block; and Highly Negative (HN), Mildly Negative (MN) or neutral in negative block. The valence and arousal of the pictures for each stimulus category were balanced according to the collected rating data of another 30 subjects (Figure 1B). In order to verify the validity of the pictures selected for each category, we chose one representative picture from high, mild and neutral categories during positive and negative blocks, respectively. Participants were asked to evaluate the emotional valence of the chosen pictures by using a self-report 9-point rating scale (ranging from 1 = “very unpleasant” to 9 = “very pleasant”). The sequence of positive and negative blocks was counterbalanced across subjects, and the sequence of standard and deviant pictures was randomized in each session. All the pictures were identical in size and resolution.

### Behavioral Procedures

**Figure 2:**
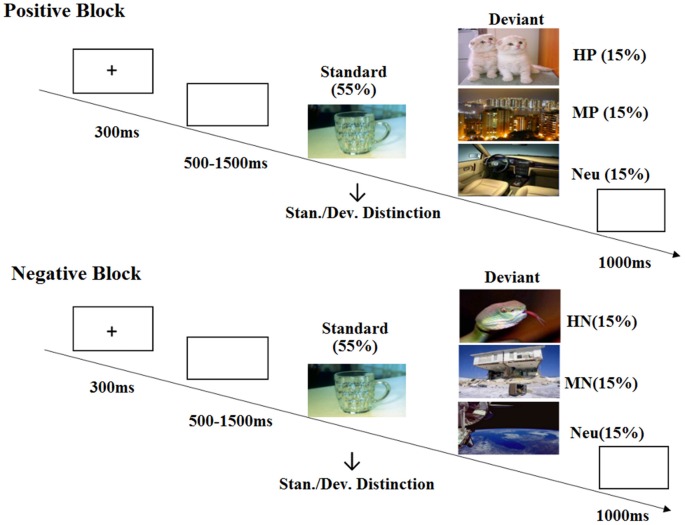
Schematic illustration of the experimental procedure and the stimulus examples.

Each trial began with a 300 ms presentation of a small black cross on the white computer screen. Then, a blank screen which lasted randomly for 500–1500 ms was presented and was followed by the onset of picture stimulus. The duration of each picture was 1000 ms. All the subjects were instructed to press the “F” key with left index finger as accurately and quickly as possible if the standard picture appeared, and to press the “J” key with the right index finger if the deviant picture appeared. After each picture, a blank screen was presented for 1000 ms (Figure 2). At the end of each session, subjects received a feedback of their task accuracy for the standard and deviant stimuli. A 2-min break was used after each session to avoid the fatigue effect. Immediately before and after either block, subjects were asked to rate their mood state by using a self-report 9-point rating scale (ranging from 1 = “very unpleasant” to 9 = “very pleasant”). A practice of ten trials was used in the beginning of the experiment, and the formal experiment did not start until they reached an accuracy rate of 90%.

### ERP Recording and Data Reduction

The EEG was recorded from 64 scalp sites using tin electrodes mounted on an elastic cap (Brain Product, Munchen, Germany), and the average of bilateral mastoids was used for offline ERP computation. The vertical EOG was monitored with electrode placed at the supra-orbital ridge of the left eye. The horizontal EOG was recorded from the left vs. right orbital rim. The EEG and EOG were amplified by using a recording bandpass of 0.01–100 Hz (FIR filter) at a sampling rate of 500 Hz. All the electrodes impedance values were below 10 kΩ.

Averaging of ERPs was computed offline using the Vision Analyzer software developed by the Brain Products Company (Munich, Germany). EEG was band-pass filtered with cutoffs between 0.1–30 Hz for offline analysis and was corrected for blinks and artifacts using the recommended method of eye movement correction algorithm (Gratton et al., 1983). The averaging epoch was 1000 ms, including a 200 ms pre-stimulus baseline. In the procedure of artifact rejection, the amplitudes exceeding ±120 μV were considered artifacts and were excluded from averaging. Lastly, all the trials adopted for ERP analysis were those with correct response.

### Statistical Analyses

**Figure 3:**
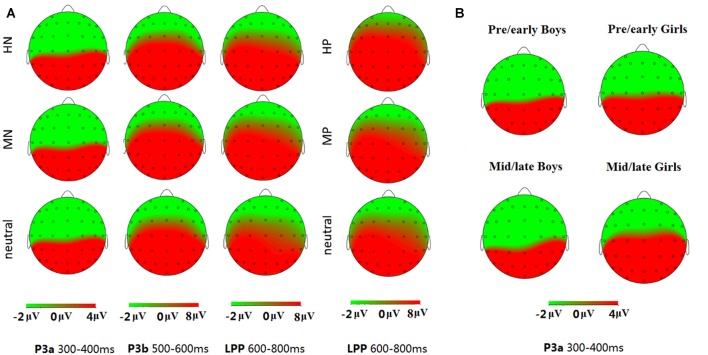
Topographical maps of the voltage amplitudes elicited by different stimulus categories in P3a (300–400 ms), P3b (500–600 ms) and late positive potential (LPP; 600–800 ms) time interval **(A)**; and topographical maps of the voltage amplitudes for the negative block in the P3a (300–400 ms) time interval for each group **(B)**.

Data analysis was focused on P3a, P3b and LPP components according to ERP morphology and priori hypotheses. Consistent with the scalp distributions in abundant ERP studies (Delplanque et al., 2005, 2006; Yuan et al., 2008), P3a shows the highest amplitudes in midline centroparietal region and P3b shows the highest amplitudes in midline parietal region, so we selected the three electrodes of CP1, CPz, CP2 for P3a analysis, and six electrodes of CP1, CPz, CP2, P1, Pz, P2 for P3b analysis. For LPP analysis, we picked three electrodes on centroparietal region (CP1, CPz, CP2), and three on parietal region (P1, Pz, P2; Figure 3A). A repeated measure analysis of variance (ANOVA) was used for the analysis of these components during positive and negative blocks, respectively, with intensity (3 levels: highly, mildly and neutral) as a repeated factor while sex and puberty as between-subjects factors. The degrees of freedom of the F-ratio were corrected for violation of spherical assumption according to the Greenhouse-Geisser method. Bonferroni-Holm method was used for *post hoc* comparisons if significant main or interaction effects appeared. The data analysis was conducted using SPSS software (version 16.0).

## Results

### Behavioral Data

In the negative block, the analysis of variance of accuracy data, with intensity as the within factor while puberty, sex as between factors, showed similar accuracy in boys and girls [*M*_boy_ = 0.94, *M*_girl_ = 0.96, *p* = 0.12, ns]. Also, the accuracy was similar across pre-/early and mid-/late pubertal stages [*M*_pre-/early puberty_ = 0.95, *M*_mid-/late puberty_ = 0.95, *p* = 0.83, ns]. No interaction effects were detected. Similarly, the analysis of reaction times detected no significant intergroup differences and interaction with emotion intensity. These results suggest that the task is suitable for all the subjects of the current study. In addition, the analysis of valence assessment data showed no other significant effects except for a main effect of emotion intensity (*F*_(2,138)_ = 46.59, *p* < 0.001), as neutral picture was evaluated significantly happier than the HN and MN pictures. The ANOVA of mood assessment data showed happier mood scores in pre-/early subjects compared to the mid-/late subjects (*F*_(1,69)_ = 8.99, *p* < 0.01; see Figure 1C).

Similarly, the ANOVA of accuracy data in the positive block showed no significant differences between boys and girls (*M*_boy_ = 0.94, *M*_girl_ = 0.96, *p* = 0.06, ns), or between pre-/early and mid-/late pubertal stages (*M*_pre-/early puberty_ = 0.94, *M*_mid-/late puberty_ = 0.95, *p* = 0.36, ns). Also, no significant interaction effects were detected. The ANOVA of RT data also showed no other effects except for longer response time for neutral pictures (595.21 ± 4.96 ms) than that of HP pictures (589.55 ± 4.97 ms; *p* < 0.05). The analysis of valence assessment data confirmed that HP pictures (*M* = 7.39) were rated more positive than MP pictures (*M* = 6.48; *p* < 0.001), which were in turn rated as more positive than neutral stimuli (*M* = 5.21; *p* < 0.001). Also, the analysis of mood assessment data showed more positive mood in pre-/early compared to mid-/late subjects (*F*_(1,69)_ = 6.41, *p* < 0.05; see Figure 1C).

### ERP Data

#### P3a (300–400 ms)

For negative valence, a repeated measures ANOVA showed a significant main effect of intensity (*F*_(2,138)_ = 5.85, *p* < 0.01), with HN stimuli (10.73 ± 1.23 μV) eliciting larger amplitudes than MN (9.34 ± 1.10 μV; *p* < 0.01) and neutral (9.28 ± 1.13 μV; *p* < 0.05) stimuli. In addition, there was a significant main effect of sex (*F*_(1,69)_ = 7.29, *p* < 0.01). Girls (12.81 ± 1.60 μV) exhibited more pronounced amplitudes than boys (6.75 ± 1.58 μV).

More importantly, there was a significant interaction effect of sex and puberty (*F*_(1,69)_ = 5.40, *p* < 0.05). As there was also a significant sex and puberty interaction in age, it is necessary to see whether this interaction effect may survive after isolating the age differences. Thus, we used an analysis of covariance model with age as a covariate, and the results continued to show a significant sex by puberty interaction in P3a amplitudes (*F*_(1,68)_ = 4.16, *p* < 0.05).

**Figure 4:**
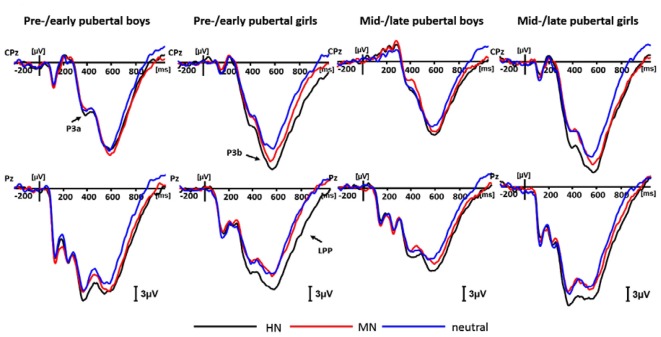
Averaged event-related potentials (ERPs) for each group during highly negative (HN; black lines), mildly negative (MN; red lines) and neutral (blue lines) conditions in the negative block.

The simple effect analysis showed no significant sex differences in response to negative pictures for the pre-/early pubertal subjects (*F*_(1,34)_ = 0.07, *p* = 0.80, ns), while girls (15.93 ± 2.17 μV) exhibited larger amplitudes than boys (4.66 ± 2.23 μV) in the mid-/late pubertal subjects, irrespective of emotion intensity (*F*_(1,35)_ = 13.13, *p* < 0.01; Figures 3B, 4). Breaking down the interaction by another direction showed similar P3a amplitudes for pre-/early and mid-/late pubertal boys (*F*_(1,35)_ = 1.42, *p* = 0.24, ns), while the P3a amplitudes were significantly enhanced for mid-/late relative to pre-/early pubertal girls (*F*_(1,34)_ = 5.11, *p* < 0.05).

For positive valence, there was a significant main effect of emotion intensity (*F*_(2,138)_ = 19.80, *p* < 0.001), HP pictures (11.70 ± 1.16 μV) elicited larger amplitudes than MP pictures (9.91 ± 1.16 μV; *p* < 0.01), which, in turn, elicited larger amplitudes than the neutral stimuli (8.78 ± 1.12 μV; *p* < 0.05). No other significant main or interaction effects were detected.

#### P3b (500–600 ms)

For negative valence, the analysis of P3b amplitudes showed a significant main effect of emotion intensity (*F*_(2,138)_ = 23.17, *p* < 0.001), and a significant emotion intensity by sex interaction effect (*F*_(2,138)_ = 3.47, *p* < 0.05). The simple effect analysis showed a significant main effect of intensity in boys (*F*_(2,72)_ = 4.59, *p* < 0.05). HN stimuli (17.78 ± 1.84 μV; *p* < 0.05), instead of MN stimuli (16.91 ± 1.71 μV; *p* = 0.19), elicited larger amplitudes than neutral stimuli (15.86 ± 1.67 μV). There was also a significant emotion intensity effect in girls (*F*_(2,70)_ = 22.18, *p* < 0.001). HN stimuli (23.03 ± 1.45 μV) elicited larger amplitudes than MN stimuli (20.53 ± 1.24 μV; *p* < 0.001), which, in turn, elicited larger amplitudes than neutral stimuli (18.77 ± 1.26 μV; *p* < 0.05; Figure 4).

For positive valence, there were no other significant main or interaction effects, except for a main effect of emotion intensity (*F*_(2,138)_ = 22.52, *p* < 0.001). HP stimuli (21.10 ± 1.18 μV) elicited larger amplitudes than MP (18.58 ± 1.13 μV; *p* < 0.001) and neutral (18.06 ± 1.06 μV; *p* < 0.001) stimuli, while the latter two conditions showed no significant differences (*p* = 0.47, ns).

#### LPP (600–800 ms)

For negative valence, there was a main effect of intensity (*F*_(2,138)_ = 40.38, *p* < 0.001), and a significant intensity by sex interaction effect (*F*_(2,138)_ = 3.38, *p* < 0.05). The following analysis showed a significant intensity effect in boys (*F*_(2,72)_ = 11.92, *p* < 0.001), with the LPP amplitudes more pronounced during HN (11.00 ± 1.53 μV; *p* < 0.001) and MN (10.14 ± 1.47 μV; *p* < 0.01) relative to neutral stimuli (8.06 ± 1.31 μV). Also, the intensity effect was significant in girls (*F*_(2,70)_ = 31.58, *p* < 0.001), with HN stimuli (14.24 ± 1.35 μV) eliciting larger LPP amplitudes than MN stimuli (11.65 ± 1.10 μV; *p* < 0.001), which in turn elicited larger amplitudes than the neutral stimuli (9.07 ± 1.15 μV; *p* < 0.001). To show sex differences more clearly, we computed an index of emotional effect as defined by the emotion-neutral differences, and then conducted a *t*-test for the emotion effect across sexes. The results showed that the emotional effect for HN stimuli was stronger in girls (5.17 ± 4.63 μV) than in boys (2.95 ± 4.27 μV; *t* = −2.13, *df* = 71, *p* < 0.05). By contrast, the size of the emotion effect for MN stimuli was similar across boys and girls (*t* =− 0.60, *df* = 71, ns; Figure 4).

**Figure 5:**
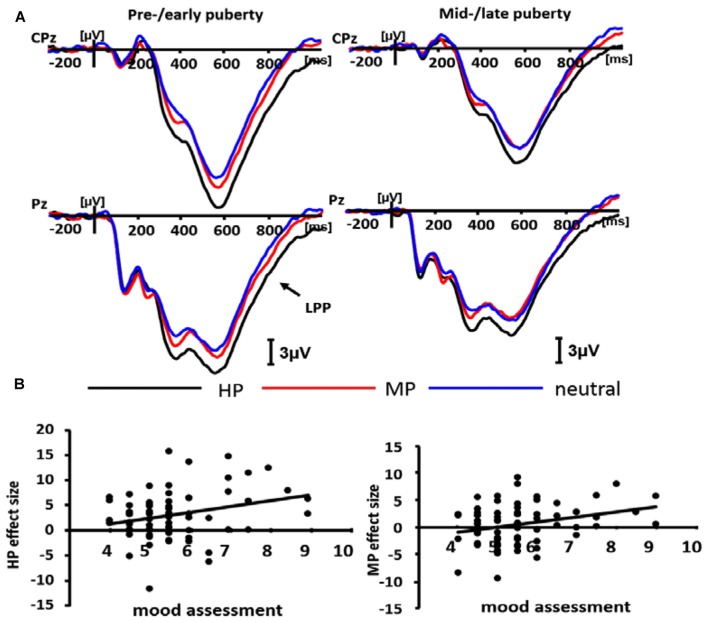
Averaged ERPs for pre-/early and mid-/late pubertal sample during highly positive (HP; black lines), mildly positive (MP; red lines) and neutral (blue lines) conditions in the positive block **(A)**, and the scatterplot for the correlation between the emotion effect for HP, MP stimuli and mood assessment data **(B)**.

For positive valence, we observed a significant main effect of intensity (*F*_(2,138)_ = 19.29, *p* < 0.001) and a significant intensity by puberty interaction in LPP amplitudes (*F*_(2,138)_ = 3.64, *p* < 0.05). Also, the significance of this puberty-related interaction was independent of age differences, as this interaction remained robust after taking age as a covariate (*F*_(2,136)_ = 4.40, *p* < 0.02). The decomposition of the interaction effect showed a significant emotion intensity effect in pre-/early group (*F*_(2,70)_ = 21.29, *p* < 0.001), with HP stimuli (14.00 ± 1.51 μV) eliciting larger amplitudes than MP stimuli (11.38 ± 1.31 μV; *p* < 0.001), which in turn elicited larger amplitudes compared to neutral stimuli (9.70 ± 1.31 μV; *p* < 0.05). By contrast, the intensity effect was not significant for mid-/late group (*F*_(2,72)_ = 3.82, *p* > 0.05, ns; Figure 5A). Therefore, the emotional arousal for positive stimuli in pre-/early adolescents was higher than that of mid-/late adolescents, and these results were consistent with the mood assessment in behavioral data.

### Correlation Analysis

A correlation analysis with Pearson method was conducted between behavioral (mood assessment) and ERP results. In LPP amplitudes of positive valence, there was a significant positive correlation between the emotion effect for HP stimuli and mood assessment (*r* = 0.275, *p* = 0.018), so was the correlation between the emotion effect for MP stimuli and mood assessment (*r* = 0.289, *p* = 0.013; see Figure 5B).

## Discussion

Prior studies have extensively investigated the profiles of brain development (both gray and white matter; Paus et al., 1999; Somerville et al., 2010) during adolescence and how physiological maturation may interact with psychosocial factors in the occurrence of adolescent psychopathology (Paus et al., 2008). Nevertheless, how pubertal development may interact with sex in modulating brains’ susceptibility to emotional stimuli and its neurophysiological correlates have yet to be systematically studied. The examination of this issue is helpful to the understanding of the sex-related prevalence of affective disturbances from adolescence. To this aim, the current study used ERP technique and manipulated the emotion intensity of positive and negative stimuli in an implicit emotional task. The results show, from behavioral and brain potential levels, that pubertal development is associated with reduced reaction to pleasant stimuli across sexes. Moreover, puberty enhanced attention bias for negative stimuli in females but not in males, though females exhibited enhanced cognitive and experiential sensitivity to negative stimuli than males, regardless of puberty.

First, irrespective of pubertal status, girls rather than boys showed significantly more pronounced P3b amplitudes for MN compared to neutral stimuli, while the emotion effect for HN stimuli in LPP amplitudes was more pronounced in girls than in boys. These results suggest that girls weigh aversive, threatening contents more heavily and elicit higher experiential arousal to these stimuli than boys, irrespective of puberty. Though there was no female preponderance in the overall prevalence of affective disorders during childhood (Nolen-Hoeksema and Girgus, 1994; Wichstrøm, 1999; Wesselhoeft et al., 2015), epidemiological studies have consistently shown more incidence of phobia-related and anxiety disorders in girls compared to boys during prepubertal, 6–9 year old childhood (Almqvist et al., 1999; Kroes et al., 2001), which are characterized by heightened responses to uncertain or actual threats (Pflugshaupt et al., 2005; Grupe and Nitschke, 2013). These evidences are consistent with our findings that girls exhibited higher P3b and LPP amplitudes for aversive stimuli than boys, irrespective of pubertal status.

Second, the results showed that pre-/early but not mid-/late adolescents exhibited enhanced LPP amplitudes for pleasant relative to neutral stimuli, irrespective of sex. The lack of pleasure and interest in response to hedonic stimuli has been proven an essential feature of depressive disorder (Dyck et al., 1994; Crawford and Henry, 2004; Sherdell et al., 2012). Consistently, there is abundant evidence showing that pubertal transition leads to a higher prevalence of depression and related behavioral disorders, irrespective of sex (Hankin, 2006; Patton and Viner, 2007). For instance, it was reported that anhedonia and psychomotor retardation symptoms of depression tend to increase and become more prevalent with the transition from childhood into adolescence (Hankin, 2006). Prospective longitudinal studies show that average levels of depressive mood and symptoms rise substantially from childhood to middle adolescence across sexes (Ge et al., 1994, 2003), due to increasing stressful life events such as academic stress, parent-offspring and other interpersonal conflicts during adolescence (Galambos and Almeida, 1992; Laursen et al., 1998; LaRue and Herrman, 2008; Quach et al., 2015). It is worth noting that the current study also observed decreased pleasant mood ratings during mid-/late relative to pre-/early adolescents, irrespective of block category and sex. All these data suggest that pubertal transition is linked with decreased pleasant emotion sensitivity, a key element that predisposes an adolescent to a depressive state (Sherdell et al., 2012).

Third, we observed higher P3a amplitudes in girls than in boys in mid-/late adolescents, and higher P3a amplitudes for mid-/late relative to pre-/early girls, during the negative block. This result is consistent with the previous finding that pubertal transition is associated with enhanced gamma oscillations for negative pictures in girls but not in boys (Yuan et al., 2014). Also, these results suggest that pubertal development brings girls a greater attention bias for negative stimuli compared with boys. This provides an explanation for the epidemiological reports of girls’ increased incidence of affective disturbances relative to boys that begins from early adolescence (Kessler et al., 1993; Nolen-Hoeksema and Girgus, 1994; Ge et al., 2001; Marcotte et al., 2002; Hyde et al., 2008). Prior studies suggested a couple of biological or psychosocial factors that mediate girls’ vulnerability to affective disorders during adolescence. For instance, the start of menstrual circle, which symbolizes pubertal transition of girls, leads girls to more fluxes in reproductive hormones than boys, and this flux increases emotional disturbances (Altemus, 2006; Ziomkiewicz et al., 2012). In addition, pubertal development is associated with a couple of psychosocial vulnerabilities that are more prominent in girls, such as gender role differentiation that stereotypes girls to be feminine (e.g., compliant and passive); increased interpersonal dependance, body image concerns and emotion-focused coping (Wichstrøm, 1999; Hyde et al., 2008). For example, recent studies show that the wellbeing of pubertal girls relies more on peer acceptance compared to that of prepubertal girls, while this pattern of puberty effect is not significant in boys (Guyer et al., 2014). These factors may contribute to our finding of different patterns of sex differences in attention bias for negative stimuli across pubertal stages.

It is worth noting that boys showed no significant puberty effect in P3a amplitudes, and pre-/early adolescents exhibited no sex differences in this component. This is consistent with prior reports of no sex differences in the occurrence of affective disorders in pre-pubertal childhood (Nolen-Hoeksema and Girgus, 1994; Wichstrøm, 1999; Marcotte et al., 2002). It has been indicated that pubertal development of boys is associated with increased social requirement of trait masculinity: to be less emotion-focused, more assertive, confident and action-oriented with physical resemblance to adult males (Wichstrøm, 1999; Hyde et al., 2008). On the other hand, there are evidences showing that emotional-expressive suppression is able to downregulate the emotional impacts of aversive events in young men but not in women, while both sexes benefit from the reappraisal strategy (Gross and John, 2003; Cai et al., 2016). This suggests that boys may benefit from more flexible choices of regulation strategies according to contextual requirements. Moreover, there is evidence that the increasing testosterone level during boys’ puberty is linked with more developed top-down control of prefrontal cortices (Stanton et al., 2009), which helps to regulate subcortical emotional inputs (Lieberman et al., 2007). Thus, though the puberty of boys is also linked with psychosocial stressors (McCabe and Ricciardelli, 2001; Hankin, 2006), the increased testosterone, masculinity intensification, and the flexibility of coping strategies may constitute compensatory factors for these risks. This also provides an explanation for why the incidence of affective disorders in adolescent boys is not as prevalent as that in adolescent girls. However, we need to acknowledge the limitation that our findings of puberty effect is based on cross-sectional comparison instead of being based on the longitudinal data, which may otherwise depict a fine-grained profile of emotional sensitivity varying as a function of puberty directly. Nevertheless, considering that emotion-related individual difference measures (e.g., anxiety, depression) were controlled across the four samples prior to the experiment, the conclusions based on the cross-sectional methods should be considered reliable.

In summary, in addition to our observation that females had enhanced cognitive and experiential sensitivity to negative stimuli than males, irrespective of puberty; the present study observed that puberty increased attentional bias for negative stimuli in girls but not in boys, and puberty reduced brains’ experiential sensitivity to pleasant stimuli across sexes. These patterns of pubertal developmental changes contribute to our understanding of the electrophysiological bases underlying the greater prevalence of affective disturbances in girls vs. boys during adolescence.

## Author Contributions

JYa and JYu designed the study. SZ conducted the experiment. JYa, SZ and JYu performed data analysis and prepared the manuscript. YLo assisted in data analysis and article revision. QL assisted in the experimental operation and data analysis. YLi and SX assisted in experimental operation. JYu supervised the whole research and wrote the article.

## Conflict of Interest Statement

The authors declare that the research was conducted in the absence of any commercial or financial relationships that could be construed as a potential conflict of interest.
